# Enhancement of intrarenal plasma membrane calcium pump isoform 1 expression in chronic angiotensin II‐infused mice

**DOI:** 10.14814/phy2.13316

**Published:** 2017-06-14

**Authors:** Hiromichi Wakui, Koichiro Sumida, Megumi Fujita, Yuta Ohtomo, Masato Ohsawa, Ryu Kobayashi, Kazushi Uneda, Kengo Azushima, Kotaro Haruhara, Keisuke Yatsu, Nobuhito Hirawa, Shintaro Minegishi, Tomoaki Ishigami, Satoshi Umemura, Kouichi Tamura

**Affiliations:** ^1^ Department of Medical Science and Cardiorenal MedicineYokohama City University Graduate School of MedicineYokohamaKanagawaJapan

**Keywords:** Angiotensin, hypertension, kidney disease, PMCA1

## Abstract

Plasma membrane calcium pump isoform 1 (PMCA1) is encoded by *ATPase plasma membrane Ca* ^*2+*^ *transporting 1* (*ATP2B1*), the most likely candidate gene responsible for hypertension. Although PMCA1 is highly expressed in the kidney, little is known about regulation of its renal expression in various pathological conditions in vivo. Our study was designed to elucidate regulation of renal PMCA1 expression in mice. We employed three mouse models for kidney disease. These were the unilateral ureteral obstruction (UUO), the remnant kidney using 5/6 nephrectomy, and chronic angiotensin II administration models. Mice were assessed for systolic blood pressure and renal injury in accordance with the damage induced in the specific model. Kidney PMCA1 mRNA levels were measured in all mice. The UUO model showed renal fibrosis but no changes in blood pressure or renal PMCA1 mRNA expression. Similarly, the 5/6 nephrectomy model exhibited declined renal function without changes in blood pressure or renal PMCA1 mRNA expression. In contrast, chronic angiotensin II administration increased albuminuria and blood pressure as well as significantly increasing renal PMCA1 mRNA and protein expression. These results suggest that renal PMCA1 has a role as one of the molecules involved in angiotensin II‐induced hypertension and kidney injury.

## Introduction

Recent advances in genetic analysis techniques have enabled genome‐wide association studies (GWASs). *ATPase plasma membrane Ca* ^*2+*^ *transporting 1* (*ATP2B1*) was associated with susceptibility to hypertension and GWASs, analyzing approximately 200,000 subjects worldwide, also indicated that *ATP2B1* is the most likely candidate gene responsible for hypertension (Yatsu et al. 2007; International Consortium for Blood Pressure Genome‐Wide Association Studies, 2011). GWASs on *ATP2B1*, conducted since 2012, repeatedly showed that this gene is related not only to hypertension but also to coronary artery disease, dyslipidemia, arteriosclerosis, and chronic kidney disease (CKD; Lu et al. 2012; Ferguson et al. 2013; Fontana et al. 2014; Heo et al. 2014). *ATP2B1* encodes the protein plasma membrane calcium pump isoform 1 (PMCA1). However, because systemic PMCA1 knockout mice exhibited several conditions, including viviparous lethality, relationships among PMCA1, blood pressure, and organopathy have not been sufficiently investigated in vivo (Okunade et al. 2004).

PMCA1 is a housekeeping gene expressed in many organs, including brain, heart, blood vessels, and kidneys (Caride et al. 1998; Strehler and Zacharias 2001). In the kidneys, PMCA1 is widely distributed in the glomerulus, proximal tubules, distal convoluted tubules, and collecting tubules. Studies on regulation of PMCA1 expression in rat kidneys reported that the amount of PMCA1 expressed by mesangial cells decreased with age (Rouse et al. 1997). However, there have been very few reports describing regulation of renal expression of PMCA1 under various pathological conditions.

This study aimed to preliminarily elucidate regulation of renal PMCA1 expression in the mouse using three, relatively unrelated, kidney disease models. These included the unilateral ureteral obstruction (UUO), 5/6 nephrectomy, and chronic angiotensin II (Ang II) administration models.

## Materials and Methods

### Animals

Male C57BL/6 mice (9–11 weeks old) were from Charles River Laboratories. Mice were housed in a controlled environment with a 12‐h light–dark cycle at a temperature of 25°C and given free access to a standard diet (0.3% NaCl, 3.6 kcal/g, and 13.3% energy as fat; Oriental MF, Oriental Yeast Co., Ltd.) and water. This study was performed in accordance with the National Institute of Health guidelines for the use of experimental animals. All animal studies were reviewed and approved by the Animal Studies Committee of Yokohama City University.

### Blood pressure measurement

Systolic blood pressure and heart rate were measured by the tail‐cuff method (BP‐monitor MK‐2000; Muromachi Kikai Co.). It is known that heat stress often causes damage to mice. The MK‐2000 BP monitor made it possible to measure blood pressure without preheating the animals and using anesthesia, thus avoiding these very stressful conditions, as described previously (Wakui et al. 2010a, 2010b). At least eight readings were taken for each measurement.

### The UUO model

The UUO procedure was performed using C57BL/6 mice, as described previously (Matsuda et al. 2013). Briefly, with mice under anesthesia, the left ureter was ligated at two locations. Mice receiving the procedure were killed under anesthesia 7 days after UUO. Control animals instead received a sham operation, in which the ureters were manipulated but not ligated and were killed under the same conditions as those receiving UUO.

### The 5/6 nephrectomy model

Mice were assigned to 5/6 nephrectomy or sham‐operated control groups. For the 5/6 nephrectomy group, a right subcapsular nephrectomy was performed, followed by surgical resection of the upper and lower thirds of the left kidney under isoflurane anesthesia, as described previously (Kobayashi et al. 2017). Control animals underwent sham procedures, including decapsulation of both kidneys.

### The Ang II treatment model

Ang II infusion was performed as previously described (Wakui et al. 2013b; Ohsawa et al. 2014). Briefly, Ang II (2000 ng/kg per minute) was infused subcutaneously into mice for 14 days using an osmotic minipump (Model 2002, ALZET, Palo Alto, CA).

### Urine collection

Metabolic cages were used to collect urine as described previously (Wakui et al. 2015). Mice were given free access to tap water and fed a standard diet.

### Biochemical analysis

Arterial blood was collected by cardiac puncture under anesthesia with an intraperitoneal injection of pentobarbital (50 mg/kg). Whole blood was centrifuged at 3000 rpm at 4°C for 15 min to separate plasma. Urinary creatinine, urinary albumin, and serum creatinine levels were assessed with an autoanalyzer (Hitachi 7180; Hitachi, Tokyo, Japan).

### Histological analysis

Histological analysis was performed as described previously (Tsurumi et al. 2006; Dejima et al. 2011). Kidneys were fixed with 4% paraformaldehyde and embedded in paraffin. Sections of 4 *μ*m thickness were stained with Masson's trichrome.

### Real‐time quantitative RT‐PCR analysis

Total RNA was extracted from renal tissue using ISOGEN (Nippon Gene), and cDNA synthesized using the SuperScript III First‐Strand System (Invitrogen). Real‐time quantitative RT‐PCR was performed using an ABI PRISM 7000 Sequence Detection System by incubating reverse transcription products with TaqMan PCR Master Mix and a designed TaqMan probe (Applied Biosystems), as described previously (Wakui et al. 2010a, 2013a; Ohsawa et al. 2014). mRNA levels were normalized to those of 18S rRNA controls.

### Membranous protein extraction and immunoblot analysis for PMCA1

For the analysis of the protein levels of renal PMCA1 expression, membranous proteins were extracted from kidney tissues using the Plasma Membrane Extraction Kit (Biovision; K268‐50) according to the manufacturer's protocol and then used for SDS‐PAGE (Wakui et al. 2013b; Ohsawa et al. 2014). The protein concentration of each sample was measured with a DC protein assay kit (Bio‐Rad) using bovine serum albumin as the standard. Equal amounts of protein extract from the tissue samples were fractionated on a 5–20% polyacrylamide gel (ATTO), then transferred to a polyvinylidene difluoride (PVDF) membrane using the iBlot Dry Blotting System (Invitrogen). Membranes were blocked for 1 h at room temperature with phosphate‐buffered saline containing 5% skim milk powder, and probed overnight at 4°C with specific primary antibodies. The membranes were then washed and incubated with secondary antibodies for 40 min at room temperature. After they were washed, the sites of the antibody–antigen reaction were visualized by enhanced chemiluminescence substrate (GE healthcare). The images were quantitated using a FUJI LAS3000 Image Analyzer (FUJI Film). Antibodies against PMCA1 (ab3528, Abcam) and *β*‐actin (A5441, Sigma‐Aldrich) were used.

### Statistical analysis

All quantitative data are expressed as means ± SE. Differences were analyzed by Student's unpaired *t*‐test for two groups or by analysis of variance (ANOVA) for multiple comparisons. Differences among groups for categorical variables were analyzed using the chi‐square test. *P*s < 0.05 were considered statistically significant.

## Results

### Effects of UUO on renal fibrosis, blood pressure, and renal PMCA1 expression

**Figure 1:**
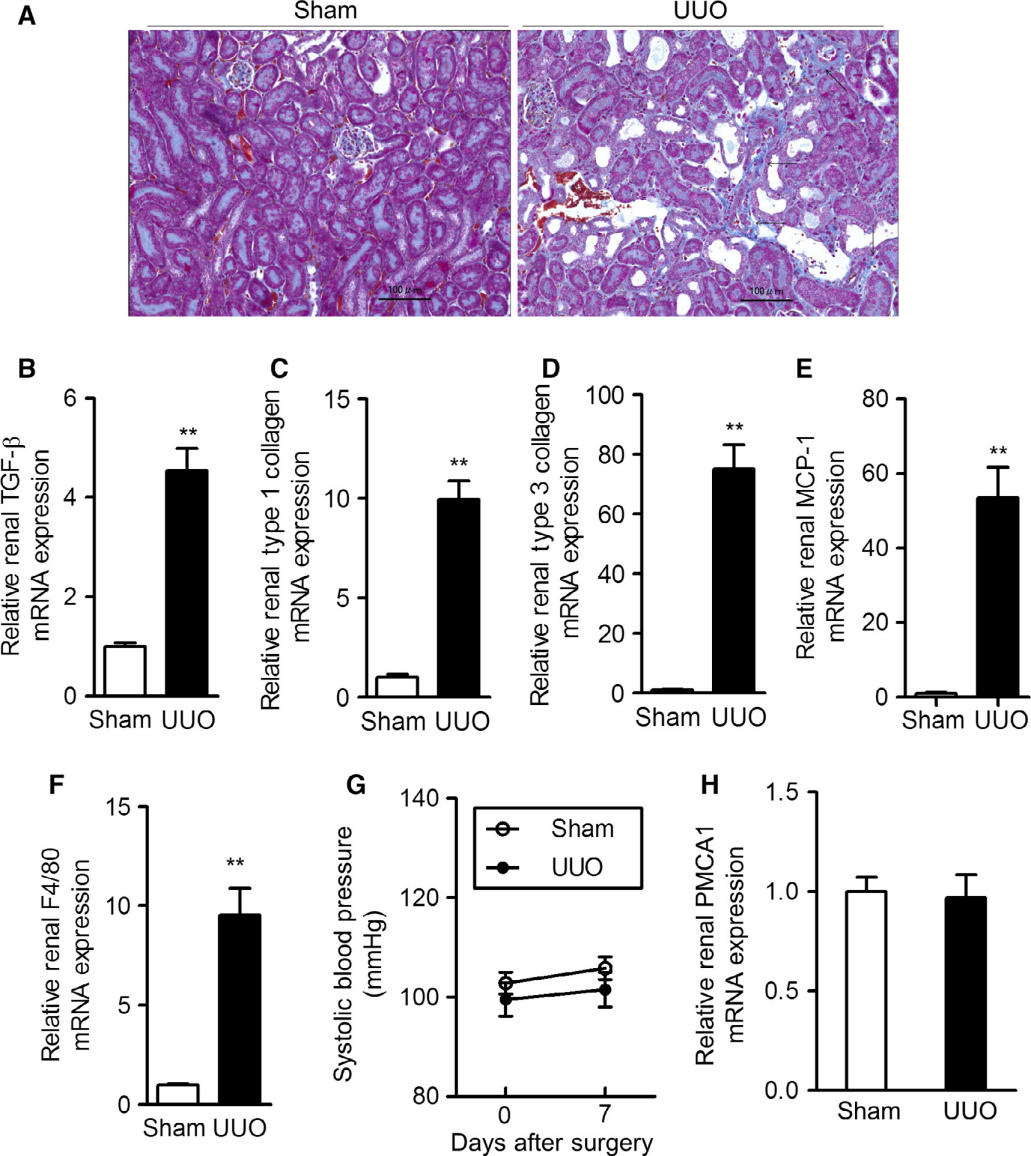
Effects of UUO on renal fibrosis, blood pressure, and renal PMCA1 expression in mice. (A) Representative images of Masson's trichrome‐stained kidney sections from mice at 7 days after sham procedure or UUO (original magnification, 200×; bar, 100 *μ*m). Arrows indicate fibrotic areas. Renal mRNA expression of fibrosis‐related factors (B, TGF‐*β*; procedure or UUO. Values are means ± SE (*N* = 7 per group). **P < 0.01 versus sham‐operated mice. (G) Time course of systolic blood pressure after sham procedure or UUO. Values are means ± SE (*N* = 7 per group). (H) Renal PMCA1 mRNA expression at 7 days after sham procedure or UUO. Values are means ± SE (*N* = 7 per group). Sham, sham procedure control; UUO, unilateral ureteral obstruction; PMCA1, plasma membrane calcium pump isoform 1.

In the first experiments, we performed UUO in mice, a procedure leading to progressive renal fibrosis, independent of systemic immune disease (Chevalier et al. 2009). At 7 days after surgery, mice undergoing UUO exhibited hydronephrosis and renal tubulointerstitial fibrosis (Fig. 1A). We next compared renal expression of fibrosis‐related genes (collagen‐1*α*, ‐3*α*, and transforming growth factor‐*β* [TGF‐*β*]) in mice undergoing UUO compared with the sham operation. Renal expression levels of collagen‐1*α*, collagen‐3*α*, and TGF‐*β* mRNA were markedly increased in mice undergoing UUO, compared with the sham operation (Fig. 1B, C, and D). Involvement of monocyte chemoattractant protein‐1 (MCP‐1), a member of the C‐C chemokine superfamily, in the pathogenesis of tissue fibrosis was reported (Wada et al. 2007). We, therefore, compared renal levels of MCP‐1 mRNA in the two groups of mice and found that MCP‐1 expression was remarkably higher in mice undergoing UUO than in sham‐operated mice (Fig. 1E). In addition, we examined mRNA expression of F4/80, markers of macrophage infiltration, in kidneys. UUO significantly increased mRNA expression of F4/80 in obstructed kidney (Fig. 1F).

Systolic blood pressure was similar in mice receiving UUO to that in sham‐operated mice (Fig. 1G). We further examined PMCA1 mRNA levels in kidneys from mice in the two groups and found no difference (Fig. 1H). These results indicated that UUO did not affect either blood pressure or renal PMCA1 expression.

### Effects of 5/6 nephrectomy on renal function, blood pressure, and PMCA1 expression

**Figure 2:**
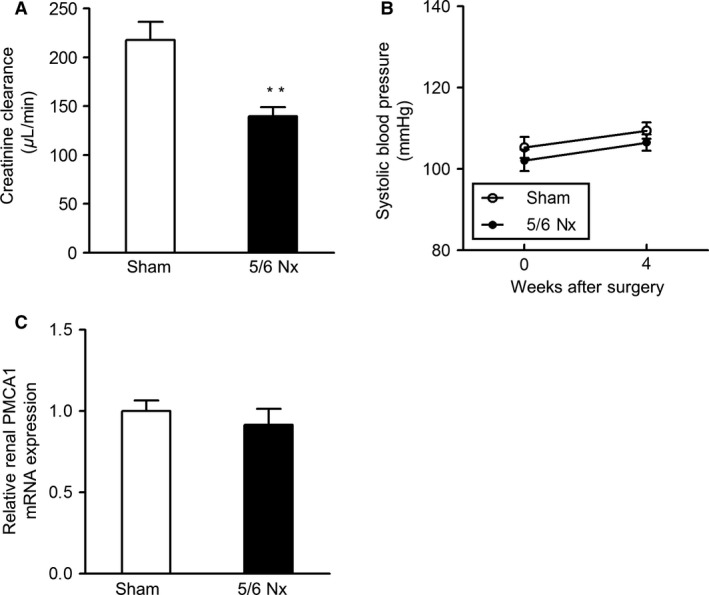
Effects of 5/6 nephrectomy on renal function, blood pressure, and renal PMCA1 expression in mice. (A) Renal function, as determined by creatinine clearance, at 4 weeks after sham procedure or 5/6 nephrectomy. Values are means ± SE (*N *= 7 per group). **P < 0.01 versus sham. (B) Time course of systolic blood pressure after sham procedure or 5/6 nephrectomy. Values are means ± SE (*N *= 7 per group). (C) Renal PMCA1 mRNA expression at 4 weeks after sham procedure or 5/6 nephrectomy. Values are means ± SE (*N* = 7 per group). Sham, sham procedure control; 5/6 Nx, 5/6 nephrectomy; PMCA1, plasma membrane calcium pump isoform 1.

In the second set of experiments, we utilized the remnant kidney model in mice, created using 5/6 nephrectomy to reduce renal mass. This procedure mimics many features of human CKD and is widely used as a model for progressive CKD (Lenihan et al. 2016). At 4 weeks after surgery, renal function, assessed using estimated creatinine clearance, was significantly decreased in mice undergoing 5/6 nephrectomy, compared with in sham‐operated mice (Fig. 2A).

Systolic blood pressure in mice undergoing 5/6 nephrectomy was similar to that in sham‐operated mice (Fig. 2B). Furthermore, PMCA1 mRNA levels were similar in mice undergoing 5/6 nephrectomy as in sham‐operated mice (Fig. 2C). These results indicated that 5/6 nephrectomy did not affect either blood pressure or renal PMCA1 expression.

### Effects of Ang II treatment on albuminuria, blood pressure, and renal PMCA1 expression

**Figure 3:**
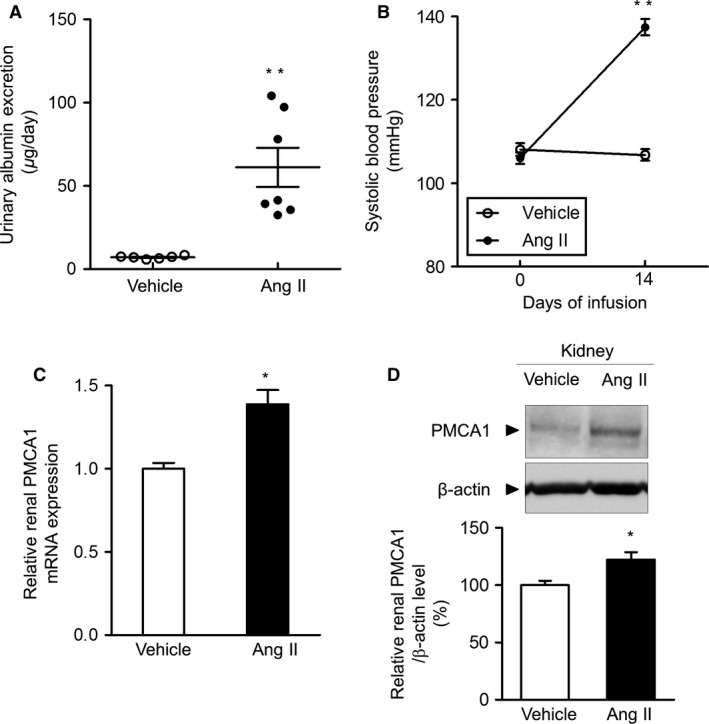
Effects of angiotensin II treatment on albuminuria, blood pressure, and renal PMCA1 expression in mice. (A) Urinary albumin excretion after administration of vehicle or angiotensin II (2000 ng/kg per min) for 2 weeks. Values are means ± SE (*N *= 6–7 per group). **P < 0.01 versus vehicle. (B) Time course of systolic blood pressure after administration of vehicle or angiotensin II. Values are means ± SE (*N* = 6–8 per group). **P < 0.01 versus vehicle. (C) Renal PMCA1 mRNA expression at 14 days after the start of vehicle or angiotensin II infusion. Values are means ± SE (*N *= 7 per group). Vehicle, vehicle‐infused mice; Ang II, angiotensin II‐infused mice. *P < 0.05 versus vehicle. (D) Renal PMCA1 protein expression at 14 days after the start of vehicle or angiotensin II infusion. Values are means ± SE (*N *= 8 per group). Vehicle, vehicle‐infused mice; Ang II, angiotensin II‐infused mice; PMCA1, plasma membrane calcium pump isoform 1. *P < 0.05 versus vehicle.

In the third set of experiments, we administered Ang II to mice for 2 weeks. In the kidney, Ang II constricts efferent more than afferent arterioles, causing increased intraglomerular pressure. Ang II also stimulates mesangial cell proliferation and extracellular matrix synthesis and directly induces intracellular junctional opening in vascular endothelial cells, promoting glomerular capillary permeability (Remuzzi et al. 2005; Wakui et al. 2014). These multiple synergistic effects of Ang II in the kidney cause progression of proteinuria. In our study, chronic Ang II infusion for 2 weeks significantly increased urinary albumin excretion (Fig. 3A). In addition, the Ang II infusion significantly increased blood pressure (Fig. 3B). Furthermore, renal PMCA1 mRNA levels were significantly higher in Ang II‐infused mice compared with vehicle‐infused mice (Fig. 3C). Similarly, renal PMCA1 protein levels were significantly increased by chronic Ang II infusion (Fig. 3D).

## Discussion

In this study, we investigated blood pressure and renal PMCA1 expression in three, relatively unrelated, kidney disease models: UUO, which induces kidney fibrosis; 5/6 nephrectomy, which causes reduced renal function; and chronic Ang II administration, which leads to albuminuria. Compared with in control mice, renal PMCA1 expression was not affected in the UUO and 5/6 nephrectomy models, but was significantly higher in the chronic Ang II administration model. To our knowledge, this is the first report revealing the regulation of renal PMCA1 expression in kidney disease models of mice. Renal expression of PMCA1, highly expressed in the kidney and regarded as a hypertension candidate gene, can be altered by chronic Ang II infusion, concomitant with the development of hypertension and albuminuria. Although there is not any data of the functional role of renal PMCA1 in the present study, the results potentially suggest that renal PMCA1 has a role as one of the molecules involved in Ang II‐induced hypertension and kidney injury.

PMCA1 acts as a channel, allowing movement of calcium ions from inside to outside the cells. We previously showed that, in vascular smooth muscle cell‐specific PMCA1‐knockout mice, a vasoactive agonist potentiated their already increased intracellular calcium concentrations, causing vasoconstriction which, in turn, led to hypertension (Kobayashi et al. 2012). Ang II stimulants generally increase intracellular calcium concentrations in a variety of cell types (Fukuta et al. 1998; Assunção‐Miranda et al. 2005; Coffman 2014; Earley and Brayden 2015). In renal tubules, Ang II binds to AT1 receptors on the cell membrane, increasing intracellular calcium concentrations and, consequently, promoting sodium reabsorption. Therefore, increased renal PMCA1 expression following chronic Ang II administration in our study might have represented an antagonistic response to elevated intracellular calcium concentrations and sodium reabsorption.

Our study did not demonstrate whether increased renal PMCA1 expression following chronic Ang II administration was the cause or result of hypertension‐related kidney injury. Thus, the causal relationship between these two phenomena remains unknown. Therefore, the functional role of PMCA1 in kidney injury and hypertension provoked by Ang II treatment must be further investigated, using in vitro renal cell lines (e.g., intracellular calcium concentration measurements/siRNAs) or animal models genetically modifying kidney‐specific PMCA1 expression.

Our study had several other limitations. We did not identify the renal cells in which changes in PMCA1 expression occurred. In addition, the molecular mechanism regulating PMCA1 expression remains unknown. Nevertheless, in our study, we investigated regulation of renal PMCA1 expression in three in vivo mouse kidney disease models. We demonstrated that, of these, chronic Ang II administration significantly increased renal PMCA1 expression, concomitant with the development of renal injury and hypertension.

## Conflict of Interest

None declared.
